# Olfactory and gustatory dysfunction caused by SARS-CoV-2: Comparison with cases of infection with influenza and other viruses

**DOI:** 10.1017/ice.2020.196

**Published:** 2020-05-05

**Authors:** Tsutomu Nakashima, Hirokazu Suzuki, Masaaki Teranishi

**Affiliations:** 1 Department of Rehabilitation, Ichinomiya Medical Treatment & Habilitation Center, Ichinomiya City, Aichi, Japan; 2 Department of Otorhinolaryngology, National Center for Geriatrics and Gerontology, Aichi, Obu, Japan; 3 Departmeny of Otorhinolaryngology, Nagoya University Graduate School of Medicine, Nagoya, Japan

*To the Editor*—Among the symptoms of SARS-CoV-2 infection (or COVID-19), olfactory or gustatory dysfunction may possibly present first or may be the only symptom.^1^ Three Japanese professional baseball players complained of smell and taste dysfunction. Although 2 of them had neither fever nor cough, a viral polymerase chain reaction (PCR) test revealed that all 3 were SARS-CoV-2 positive (*The Chunichi Newspaper*, March 27, 2020). Two nurses working in the National Cancer Center Hospital underwent the viral PCR test because they had similar symptoms, and they were both SARS-CoV-2 positive, although they had neither fever nor cough (*Asahi Shimbun* newspaper [digital], March 28, 2020).

Olfactory dysfunction is caused by blockage of the nasal airways or disturbance of the sensory system, including olfactory receptor cells, and the nervous system. As the olfactory receptor cells adjoin the upper part of the nasal cavity, the receptor cells are vulnerable. Viral infection was the most common cause of loss of olfactory function. With this viewpoint, we reviewed the available literature on olfactory and gustatory dysfunction caused by influenza and other viruses. Postviral infection olfactory dysfunction was more common in women and elderly people.^2-4^ The influenza and parainfluenza type 3 viruses were reported to be causative of olfactory loss most frequently. Seasonal changes in the incidence of olfactory loss have been reported with respect to influenza and parainfluenza type 3 infections, occurring most frequently in winter and spring, respectively.^2,5^ Flanagan et al^6^ reported that the proportion of persons who received influenza vaccination was significantly lower among those with olfactory dysfunction than that in a control group. However, the adverse effect of olfactory dysfunction due to influenza vaccination was also reported. Dotty et al^7^ attributed 9 of 4,554 patients (0.19%) with olfactory dysfunction to influenza vaccination. Suzuki et al^8^ confirmed the presence of various viruses in the nasal discharge of patients with postviral infection olfactory dysfunction, such as rhinovirus, parainfluenza virus, Epstein-Barr virus, and coronavirus. Significant recovery was not observed after 24 weeks in almost all of the patients.^8^ In contrast, olfactory dysfunction due to hepatitis virus was recovered within 6 weeks in almost all cases.^9^ In acute viral hepatitis, hyposmia, dysosmia, and dysgeusia are common symptoms. As smell and taste are closely associated; persons with olfactory dysfunction and normal gustatory function often complain that they “cannot taste coffee.”^9^

Some recent reports described early improvement of olfactory and gustatory dysfunction in many COVID-19patients. According to the newspaper, olfactory and gustatory function in the professional baseball players also returned to normal relatively soon. However, only short-term follow-up investigation has been conducted regarding the effect of SARS-CoV-2 infection on the chemosensory function. Hwang^10^ reported that anosmia induced by SARS-CoV continued for >2 years in a 27-year-old woman. We believe that epidemiological investigation is required regarding the effect of SARS-CoV-2 on the olfactory and gustatory functions in terms of the frequency, time course, and relationship with other symptoms.
